# Record of Tests of Saliva

**Published:** 1874-09

**Authors:** Geo. H. Cushing

**Affiliations:** Chicago.


### ARTICLE II.

### *Record of Tests of Saliva.*

BY GEO. H. CUSHING, CHICAGO.

Read Before the Illinois State Dental Society.

I desire to present to the attention of the society, a record of certain examinations I have recently made, of the fluids of the oral cavity. I wish to preface this record by saying that I do not claim that these experiments are conclusive, or exhaustive, but on the contrary, that they are only the beginning of a series of experiments, which I hope may be sufficiently extended by others, as well as myself, to enable us bye and bye to announce some definite and reasonable conclusion therefrom.

The tests with the most striking results were made in the mouth of a young lady of about 24 or 25 years of age, sanguine-bilious temperament, dark hair and eyes, and of very fair clear skin. She enjoys excellent health, and is especially free from anything like dyspepsia. Her mouth is a particularly healthy one, with beautifully colored and healthy gums. Her teeth are beautifully shaped, small, and of pearly white color. She has lost five, four molars and one bicuspid, and at the time I commenced my observations, only three had been filled, two of which had lost the vitality of the pulps.

It could hardly be said that decay was at all actively progressing, as I only found four slight cavities that required filling, and there was every evidence that these had been a long time in reaching their present condition.

With the exception of those that had been filled, and those that I treated (all of the latter superior molars and bicuspids,) the teeth were as perfect as any I have ever examined—free, even from the suggestion of imperfection or decay.

The importance and value of these experiments, I think will be clearly apparent, when it is considered that this is only the unit in the series which it is hoped may some day be fully completed.

The tests were made with litmus paper, prepared by Squibb. The tests were applied to all parts of the mouth, the special points of observation having been, at the ducts of Steno; under the tongue; upon the forward part of the tongue and its base; and between the teeth. The latter point of observation will not be particularly referred to in the record, as it was almost uniformly neutral in its effect upon the litmus.

*Dec.* 27.—3 P. M. Fasting since morning, very marked acid reaction in every part of the mouth.

*Dec.* 29.—1 P. M. Just after eating of pickles—barely a trace of acid, hardly enough to detect, and that only between the lower teeth.

*Dec.* 30.—Just before lunch, less acid than on 27th—everywhere apparent—somewhat more so at the ducts of Steno than elsewhere. Just after lunch, much less reaction than before eating, but found everywhere as before. 3:30, same as last.

*Dec.* 31.—10 A. M., and also just before lunch, about same as on 20th at same hours. Thirty minutes after lunch a marked difference—only just apparent, except above the ducts of Steno, and very much less there than before eating.

*Jan.* 2.—10 A. M. Reaction same as before as to locality, but slightly less in degree than upon former examination at the same hour. Just before lunch, somewhat more acid. Just after lunch (with a pickle,) considerably less acid and a half hour after, having meanwhile eaten honey—still less reaction.

*Jan.* 3.—10 A. M. Greater in degree than yesterday—same as to locality, except that under tongue, two trials gave no reaction and six or eight gave very marked reaction. Just before lunch, same as at 10 A. M.,—under tongue—first test gave no reaction, six others all marked acid—after lunch, very little less reaction than before—had pickle and honey for lunch. 4 P. M. Same as last.

*Jan.* 5.—10 A. M. About same as on 3d. 10:20—having just eaten a few peppermint drops, reaction only just perceptible, except at base of tongue and on the soft palate where reaction was strong, as before eating the peppermints. 3 P. M.—Having eaten no lunch, but more or less peppermint drops during the day, reaction same as at 10 A. M., except that under the tongue it was scarcely perceptible.

*Jan.* 6.—10 A. M. Quite acid everywhere. Just before lunch, very slight generally, and much less on posterior of tongue than ever before observed. After lunch of cranberry pie and honey, very slight reaction generally, except at Steno's ducts, where it was moderate; under the tongue very slight and at back of the tongue very great.

*Jan.* 8.—10 A. M. Sick the day before with headache and ate nothing all day. Much less reaction generally than usual, especially noticeable at back of tongue. 1 P. M. Still less reaction than at 10 A. M., and back of tongue scarcely a trace.

*Jan.* 9.—10 A. M. Less than usual; 12 M. and 3 P. M. about the same.

*Jan.* 10.—10 A. M. Reaction uniform as to locality, but very slight. 1 P. M. Slightly increased; 3 P. M., somewhat more than at 1 P. M., except at base of tongue, scarcely a trace.

*Jan.* 12.—9:30 A. M. Greater reaction than on the 10th; lunch less than at 9:30.

*Jan.* 13.—10 A. M. At Steno's ducts acid reaction as great as ever observed. Under the tongue less than before, comparatively; at base of tongue barely a trace. After lunch (with pickle) very slight reaction indeed at Steno's ducts; less under tongue; none anywhere else.

*Jan.* 14.—3 P. M. Has not eaten since morning: been suffering all day with severe headache. Not a trace discernible at left parotid, and only the very slightest at right parotid; absolutely neutral elsewhere.

*Jan.* 15.—10 A. M. Moderate reaction generally. 3 P. M. Scarcely perceptible.

*Jan.* 17.—11:30 A. M. Alternately acid and neutral in some places all over the mouth. This peculiarity most marked under the tongue, where a dozen or more tests, following immediately one after the other, gave alternately very acid reaction, and absolutely neutral.

*Jan.* 21.—1 P. M. No lunch. Slightly acid at Steno's ducts and a little under tongue. 3 P. M. Slightly less than at 1 P. M., but more so at base of tongue.

*Jan.* 22.—11 A. M. Slight reaction generally, but very marked at base of tongue. 4 P. M. Quite uniform throughout the mouth, and about same degree as in morning.

*Jan* 23.—10 A. M. Slight reaction generally, and a little variable under tongue.

*Jan.* 24.—10 A. M. Moderately acid, but very uniform throughout the mouth. 3 P. M. Just after eating freely of pickles absolutely no acid reaction anywhere, except at base of tongue.. Just a perceptible alkaline reaction at the left parotid and under tongue.

*Jan.* 26.—10 A. M. Moderately acid everywhere. After lunch ( with pickles ) not a trace to be seen, except slight at left parotid.

*Jan.* 27.—10 A. M. Same as last, at same hour; no lunch or pickles. 3 P. M. Scarcely a trace of acid.

*Jan.* 29.—3 P. M. No lunch. More reaction at Steno's ducts than in the last two experiments. None under tongue nor at its base.

*Jan.* 31.—12 M. No lunch. Uniformly slight reaction everywhere. 2 P. M. Having eaten candy and nothing else, just the same as at 12 M.

*Feb.* 2.—10 A. M. Hardly a trace to be discovered. After lunch ( with pickles ) a slight reaction at parotids and no where else.

*Feb.* 10.—2 P. M. Having eaten considerable candy, very slight reaction anywhere, hardly perceptible.

We have here a record of observations made on twenty-five days, between December 27th and February 10th, most of them taken at three different hours of the day, and generally with two to five tests at every point examined made at each time of experiment. The results are before you in all their variety.

The following record has less interest, as presenting very little variety of result, but is one of the series necessary to complete our study. The record is of observations in my own mouth.

*Dec.* 31.—Alkaline reaction in several places in mouth.

*Jan.* 2.—Before and after lunch neutral.

*Jan.* 3.—10 A. M., neutral; just before lunch just a perceptible acid reaction; half an hour after lunch quite decided acid everywhere.

*Jan.* 5.—10 A. M., having eaten a few peppermint drops; and reaction greater than ever before, but not very great anywhere; most marked opposite parotid and not perceptible under tongue.

*Later.*—Less marked acid reaction.

*Jan.* 6.—Before lunch, very slight, and opposite left parotid; not perceptible elsewhere.

*Jan.* 7.—About same as 6th.

*Jan.* 8.—Just perceptible acid reaction in two or three spots, none elsewhere.

*Jan.* 9.—Slightly acid.

*Jan.* 10.—A little more than on 9th.

*Jan.* 12.—10 A. M., greater acid reaction than I have ever observed; after lunch about same.

*Jan.* 13.—Morning and after lunch, alike slightly acid.

*Jan.* 14.—3 P. M., having lunched not a trace; neutral.

*Jan.* 17.—11:30 A. M., very slight acid at left parotid; neutral elsewhere.

*Jan.* 21.—Scarcely a perceptible trace of acid anywhere or at any hour.

*Jan.* 22.—At 1 P. M. and at 4 P. M., absolutely neutral.

*Jan.* 24.—At 10 A. M. and 3 P. M., neutral.

*Jan.* 29.—More acid than I often find.

*Jan.* 31.—Neutral all day.

*Feb.* 2.—Neutral all day.

*Feb.* 10.—Neutral all day.

In submitting the above record, I have no comments to make at present, nor theories to advance. I only desire to draw the earnest attention of the profession to this subject, and to enlist as many as possible into the ranks of the observers in this direction.

The conditions and peculiarities of the secretions of the mouth are probably very imperfectly understood, and the importance of arriving, if possible, at a definite conclusion regarding them, is too evident to need any enforcement of argument.

If we would arrive at satisfactory conclusions, we can only do so by conducting, systematically, experiments of the nature of those here recorded. Of course these observations only tend to the elucidation of certain points, not of all by any means, which it is necessary for us to determine, but, when these are settled, then we can pursue our investigations in other channels.

I wish to say a few words as to the methods of making these tests.

Too great care cannot be exercised in such experiments, and for them to be of any value they must be conducted upon some general plan, so that the observations of one man may be compared with those of all others, upon the same basis.

*First*—It is very important that the same quality of litmus should be used—and probably there is none prepared more reliable as to its uniformity and delicacy than Squibb's. I would suggest that all who are disposed to assist in these investigations use Squibb's litmus exclusively.

*Second.*—The experimenter should be provided with the red as well as the blue test paper, and should test for alkalinity, as well as for acidity.

*Third.*—In making the tests, great care should be observed to have everything clean which comes in contact with the litmus, and especially it should never be used with the naked fingers, as the moisture from the hand will generally occasion an acid reaction.

The litmus, cut into small pieces, should be applied with a pair of pliers which have been previously thoroughly cleaned, then your observations will be of some real value.

Unless these precautions are observed, and uniformity of method be practiced, it is evident that such investigations can not be successful.

---

